# Effectiveness and cost-effectiveness of four different strategies for SARS-CoV-2 surveillance in the general population (*CoV-Surv Study*): study protocol for a two-factorial randomized controlled multi-arm trial with cluster sampling

**DOI:** 10.1186/s13063-021-05619-5

**Published:** 2021-09-26

**Authors:** Andreas Deckert, Simon Anders, Manuela De Allegri, Hoa Thi Nguyen, Aurélia Souares, Shannon McMahon, Matthias Meurer, Robin Burk, Matthias Sand, Lisa Koeppel, Lena Maier Hein, Tobias Roß, Tim Adler, Tobias Siems, Lucia Brugnara, Stephan Brenner, Konrad Herbst, Daniel Kirrmaier, Yuanqiang Duan, Svetlana Ovchinnikova, Kathleen Boerner, Michael Marx, Hans-Georg Kräusslich, Michael Knop, Till Bärnighausen, Claudia Denkinger

**Affiliations:** 1 Heidelberg Institute of Global Health, University of Heidelberg, Im Neuenheimer Feld 324, 69120 Heidelberg, Germany; 2 Center for Molecular Biology Heidelberg (ZMBH), University of Heidelberg, Im Neuenheimer Feld 282, 69120 Heidelberg, Germany; 3 GESIS Leibniz-Institute for the Social Sciences, B2/1, 68159 Mannheim, Germany; 4 Division of Clinical Tropical Medicine, University of Heidelberg, Im Neuenheimer Feld 324, 69120 Heidelberg, Germany; 5 Division of Computer Assisted Medical Interventions (CAMI), German Cancer Research Centre (DKFZ), Im Neuenheimer Feld 223, 69120 Heidelberg, Germany; 6 Institute for Applied Mathematics, University of Heidelberg, Berliner Str. 41-49, 69120 Heidelberg, Germany; 7 evaplan GmbH at the University Hospital, Ringstr.19b, 69115 Heidelberg, Germany; 8 Department of Infectious Diseases, Virology, University of Heidelberg, Im Neuenheimer Feld 267, 69120 Heidelberg, Germany

**Keywords:** COVID-19, Cluster-randomised controlled trial, Protocol, Population-based surveillance, Cost-effectiveness, Implementation, Pandemic

## Abstract

**Background:**

To achieve higher effectiveness in population-based SARS-CoV-2 surveillance and to reliably predict the course of an outbreak, screening, and monitoring of infected individuals without major symptoms (about 40% of the population) will be necessary. While current testing capacities are also used to identify such asymptomatic cases, this rather passive approach is not suitable in generating reliable population-based estimates of the prevalence of asymptomatic carriers to allow any dependable predictions on the course of the pandemic.

**Methods:**

This trial implements a two-factorial, randomized, controlled, multi-arm, prospective, interventional, single-blinded design with cluster sampling and four study arms, each representing a different SARS-CoV-2 testing and surveillance strategy based on individuals’ self-collection of saliva samples which are then sent to and analyzed by a laboratory. The targeted sample size for the trial is 10,000 saliva samples equally allocated to the four study arms (2500 participants per arm). Strategies differ with respect to tested population groups (individuals vs. all household members) and testing approach (without vs. with pre-screening survey). The trial is complemented by an economic evaluation and qualitative assessment of user experiences. Primary outcomes include costs per completely screened person, costs per positive case, positive detection rate, and precision of positive detection rate.

**Discussion:**

Systems for active surveillance of the general population will gain more importance in the context of pandemics and related disease prevention efforts. The pandemic parameters derived from such active surveillance with routine population monitoring therefore not only enable a prospective assessment of the short-term course of a pandemic, but also a more targeted and thus more effective use of local and short-term countermeasures.

**Trial registration:**

ClinicalTrials.gov DRKS00023271. Registered November 30, 2020, with the German Clinical Trials Register (Deutsches Register Klinischer Studien)

## Background

### Study background

In 2020, the novel coronavirus SARS-CoV-2 led to the global spread of the coronavirus disease (COVID-19), which can cause a highly fatal severe acute respiratory syndrome (SARS). In Germany, SARS-CoV-2 infections first occurred in localized hotspots in early spring, but soon spread exponentially to the larger population, with further spread contained by a 2-month-long nationwide lockdown soon after [1]. While effectively containing the virus spread and thus protecting the health of the public, this lockdown also produced long-lasting negative effects within the economic sector by increasing unemployment, bankruptcies, private and public financial debt.

A second SARS-CoV-2 wave reached Germany in fall 2020, characterized by more hotspots than in the first wave, and a fast spread of the virus across different population groups. Although inevitable to ensure disease prevention and functionality of the healthcare system, the discussion of a second nationwide lockdown was faced with strong political and societal criticism, which led initially to milder measures with respect to limiting social encounters when compared to the first wave.

Germany, as most other countries, relies largely on passive monitoring strategies with regard to SARS-CoV-2 surveillance. This usually includes a monitoring focus on those individuals presenting with clinical COVID-19 symptoms and the tracing of contact persons in case of a positive test result. To effectively monitor virus spread and to reliably predict the course of an outbreak, an active surveillance strategy may improve the monitoring and prediction of the spread of an outbreak. These include monitoring of the entire population, hence identifying and observing also asymptomatic SARS-CoV-2 carriers. Such asymptomatic carriers are estimated to represent about 40% of the population during a pandemic and therefore play a key role in uncontrolled infection spread [2, 3].

In contrast to passive surveillance, active routine surveillance further produces more reliable as well as sufficiently dynamic prevalence estimates for both asymptomatic and symptomatic virus carriers within a defined population. Such dynamic prevalence measures are crucial to not only allowing more accurate predictions on the course of a pandemic, but also in providing policy makers with essential information on containment measures that are more locally tailored and overall, less invasive. The World Health Organization therefore recommends that countries perform active surveillance, including testing, isolating cases, and tracing contacts [4]. Further, pooled sample approaches to mass testing might substantially reduce the number of tests required to screen a population and therefore represent a potential active surveillance strategy [5]. However, evidence on the application of such comprehensive, rapid and cost-effective, localized mass testing strategies in a real-world context is currently unavailable.

### Study aims

This trial therefore intends to generate evidence on the effectiveness and cost-effectiveness of different context-specific pooled sample active surveillance strategies. From the perspective of health policy makers, a key aspect of active population surveillance is to identify the most appropriate strategy with respect to its effectiveness and cost-effectiveness in the context of a health system’s defined testing capacity. The aim of the *CoV-Surv Study* is therefore to evaluate four different active surveillance strategies for their respective effectiveness in determining and predicting the prevalence of SARS-CoV-2 infections in a defined population. To also address any economic considerations, the *CoV-Surv Study* also assesses the costs and cost-effectiveness of each of the four strategies, compared to the status quo of passive surveillance. In addition, the *CoV-Surv Study* also includes an additional qualitative component to determine the acceptability of each strategy within the participating population to gain further insight into the overall applicability and user-friendliness of each strategy.

### Related literature

The novel coronavirus SARS-CoV-2 appeared first in Wuhan, China, in December 2019 and was characterized by the clustered occurrence of viral pneumonia cases [6]. Since then, the virus has spread globally with more than 70 million identified cases and more than 1.6 million deaths worldwide [7]. This rapid spread prompted the World Health Organization to declare the initial outbreak a global pandemic on March 11, 2020 [8].

Symptoms of SARS-CoV-2 infection range from fever, headache, and cough to severe shortness of breath with signs of respiratory failure [6, 9]. SARS-CoV-2 shows relatively high transmissibility during the early course of an infection in both symptomatic and asymptomatic patients [2, 10]. Its relatively high human-to-human transmission contributes not only to the pandemic spread but also creates enormous challenges to any monitoring and containment efforts. Until the global availability of effective vaccines, containment of the pandemic will largely depend on efforts preventing transmission and the strengthening of public and clinical health infrastructure [11].

The gold standard for laboratory-based SARS-CoV-2 testing includes the analysis of swabs taken from the upper respiratory tract using real-time reverse transcriptase-polymerase chain reaction (RT-PCR), a technology used to detect viral RNA [12]. This RT-PCR test is highly sensitive in detecting SARS-CoV-2 RNA during an acute infection [12]. Presently, the cost of an RT-PCR test ranges between € 25 and 40 (cost of sample collection not included), which would make an active monitoring strategy with routine laboratory-based mass testing of respiratory samples only feasible if relying on pooled testing [5].

With respect to mass testing, the collection of nasopharyngeal or oropharyngeal swabs by medically trained personnel, the required infection protection measures would pose an additional logistical challenge. A more feasible alternative sampling strategy is therefore to have individuals obtain their own swabs and sent them to the laboratory or testing site. There is also increasing evidence that simple saliva, sputum, or throat samples produced from gargling are suitable testing materials with respect to sensitive SRAS-CoV-2 detection [13–16]. Furthermore, tests based on reverse transcriptase loop-mediated isothermal RNA amplification (RT-LAMP) assays represent suitable alternatives to RT-PCR and could further reduce the cost of viral RNA testing of alternative sample materials [17, 18].

### Significance of study

Findings of the *CoV-Surv Study* will inform the choice of the most effective, acceptable, and cost-effective strategy for SARS-CoV-2 screening and testing, with the most effective and cost-effective strategy expected to be incorporated into the local public health department’s routine health surveillance activities. Additional investigation of its everyday performance will provide insight in the strategy’s applicability to real-time prevalence prediction and the usefulness of the resulting information for local policy makers to implement countermeasures to effectively prevent future nationwide lockdowns.

## Methods/design

### Study outcomes

Identification of the one best strategy will be determined by a series of parameters. Primary study outcomes include cost per correctly screened person (including costs related to contacting, pre-screening, and testing of participants); cost per positive symptomatic and asymptomatic case (including costs related to contacting, pre-screening, and testing of participants); positive detection rates (i.e., 4-week cumulative SARS-CoV-2 prevalence); and the precision of positive detection rates (i.e., confidence intervals of the estimators).

Further, secondary outcomes include the overall participation rate (i.e., number of participants for whom a test result—with and without pre-screening—is available out of the total number of contacted participants), costs per asymptomatic case, prevalence estimates, number of asymptomatic cases by strategy, ratio of symptomatic to asymptomatic cases by strategy, and participant satisfaction.

Lastly, the *CoV-Surv Study* includes additional study components, i.e., a cost-effectiveness analysis, a prognostic modeling, and a qualitative assessment of participant perception and experience, which will complement trial data with data from other sources to address the following tertiary outcomes: cost-effectiveness of each of the four active strategies compared to the status quo of passive surveillance, the development of a prognostic model to predict hospital utilization caused by SARS-CoV-2, time between test shipment and test application, and time between test shipment and test result, as well as the perception and preferences of persons in respect to being subjected to surveillance.

### Study design

**Fig. 1:**
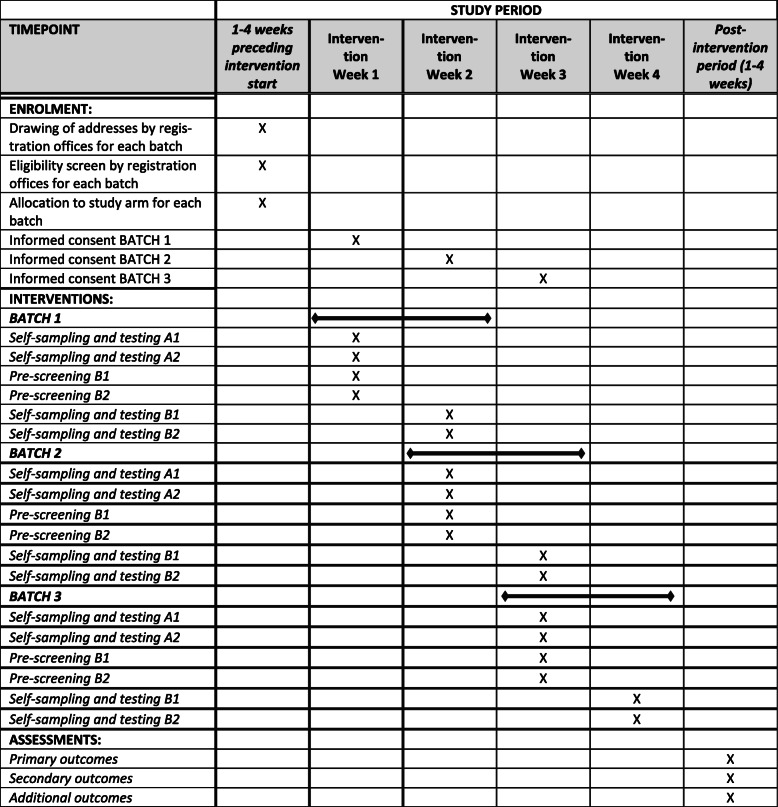
Schedule of enrolment, interventions, and assessments

The trial component of the *CoV-Surv Study* is designed as a two-factorial, randomized, controlled, multi-arm, prospective, interventional, single-blinded trial with cluster sampling and four study arms, each representing a different SARS-CoV-2 testing and surveillance strategy. Figure 1 outlines the schedule of enrolment, interventions, and assessments.

#### Strategy A1

Individuals (one per household) receive information and study material by mail with instructions on how to produce a saliva sample and how to return the sample by mail. Once received by the laboratory, the sample is tested for SARS-CoV-2 using RT-LAMP.

#### Strategy A2

Individuals (one per household) receive information and study material by mail with instructions on how to produce their own as well as saliva samples from each household member and how to return these samples by mail. Once received by the laboratory, the samples are tested for SARS-CoV-2 using RT-LAMP.

#### Strategy B1

Individuals (one per household) receive information by mail on how to complete a brief pre-screening questionnaire which asks about COVID-19-related clinical symptoms and risk exposures. Only individuals whose pre-screening score crosses a defined threshold will then receive additional study material by mail with instructions on how to produce a saliva sample and how to return the sample by mail. Once received by the laboratory, the saliva sample is tested for SARS-CoV-2 using RT-LAMP.

#### Strategy B2

Individuals (one per household) receive information by mail on how to complete a brief pre-screening questionnaire which asks about COVID-19-related clinical symptoms. Only individuals whose pre-screening score crosses a defined threshold will then receive additional study material by mail with instructions on how to produce their own as well as saliva samples from each household member and how to return these samples by mail. Once received by the laboratory, the samples are tested for SARS-CoV-2 using RT-LAMP.

Of the four surveillance strategies, strategy A1 is considered the gold standard for prevalence estimation and thus used to determine the bias introduced by each of the other strategies. To determine the cost-effectiveness, each strategy is compared to a status quo, defined as the currently practiced passive surveillance approach. In each strategy, RT-LAMP-positive samples are additionally analyzed with real-time quantitative RT-PCR (RT-qPCR) in order to minimize the number of false positives.

### Study setting

The *CoV-Surv Study* is conducted in the city of Heidelberg and the neighboring Rhein-Neckar district located in the Southwest of Germany. The combined catchment area includes approximately 700,000 inhabitants. During the first pandemic wave in May 2020, the 4-week cumulative SARS-CoV-2 prevalence of symptomatic cases for the study population was about 0.25%.

### Characteristics of participants

Eligible are individuals age 7 years or older living in the Rhein-Neckar District or Heidelberg who consented (by a legal guardian in case of minors) to provide a self-collected saliva sample (all study strategies) after completion of a brief questionnaire (strategies B1 and B2 only). For the qualitative component complementing the trial, different samples of trial participants and non-participants (i.e., eligible persons who refused to participate) are identified for an additional interview. For these interviews, only individuals age 18 years or older are eligible.

### Study materials

Participants are asked to provide a saliva sample in a testing tube after gargling with 5ml saline solution. This sample is shipped to the laboratory by mail and then analyzed for SARS-CoV-2 using the RT-LAMP test. All positive-tested samples undergo additional confirmation analysis using the RT-PCR test. Depending on the strategy, some participants first take part in an online pre-screening survey. This short survey contains between 10 and 15 questions assessing the presence of symptoms of an early SARS-CoV-2 infection. This survey also collects information on a participant’s socio-demographic background (i.e., education, employment, household size). Participants of the qualitative study component further take part in a qualitative survey or an interview with open-ended questions. Participation in this qualitative study will commence after an individual’s trial participation has been completed.

To estimate the cost of the four surveillance strategies, an activity-based micro-costing approach is used to prospectively estimate the average cost per case completely screened and per case detected for each strategy. Data on the resource consumption of all activities related to the implementation of the four strategies will be categorized as either start-up or implementation cost. For each activity, data on resource consumption for both capital cost items (e.g., building, equipment, and vehicles) and recurrent cost items (e.g., personnel, materials, and consumables) will be collected directly from respective trial records. Unit costs will be taken from secondary data sources, such as financial statements, invoices, and salary schedules.

### Study-related processes

Participants assigned to strategy A1 are initially contacted by postal mail and receive an information letter outlining and describing the study purpose, the study procedure, the consent processes, and a declaration of consent. This initial letter also contains contact information of the study-specific hotline provided with the support of the local health department. The materials for self-sampling (including a testing tube with a unique barcode identification number) together with a stamped return envelope and instructions on how to return the sample as well as the consent form are also provided in this initial letter. The information letter further contains a link to an online video that provides instructions for the self-sampling procedure. In strategy A1 completion of a short SARS-CoV-2 symptoms survey also provided to each participant is voluntary.

Participants assigned to strategy A2 receive in addition to the above testing materials for up to three of their household members and instructions on how to order additional testing materials via the hotline if necessary. The cover letter also includes additional information on the declaration of consent for each participating household member. Completion of the SARS-CoV-2 symptoms survey is voluntary for each household member.

Participants assigned to strategy B1 are initially contacted by postal mail and receive an information letter outlining and describing the study purpose, the study procedure, the consent processes, and a declaration of consent. This initial letter also contains contact information of the study-specific hotline provided with the support of the local health department. Prior to receiving testing materials, these participants are asked to consent to and complete an online (smartphone, tablet, and computer-compatible) SARS-CoV-2 symptom pre-screening survey (to be completed by a legal guardian in case of minors). A paper-based back-up option for pre-screening is provided to participants without online access. Only participants with a positive result based on the pre-screening will receive testing materials by postal mail.

Participants assigned to strategy B2 are in addition to the above asked to conduct the initial pre-screening with all of their household members and will then receive testing material for those individuals in the household with a positive screening result.

All tested participants are able to query their test results online using the unique identification number assigned to them. In cases with a positive RT-LAMP test, participants are further informed about the additional RT-qPCR confirmation test. Once this test confirms a SARS-CoV-2 infection, the local health department is informed about the case and the participant will be directly contacted by the health department for additional routine contact tracing.

Participants of the qualitative component are contacted either by mail or phone and receive an information letter outlining and describing the purpose of this quality study component and related consent processes.

### Randomization process

The total study sample consists of 10,000 participants with each strategy enrolling 2500 randomly selected participants. Participants are identified and sampled by each selected municipality’s population register provided by the respective registration office. Individuals or households are drawn as a stratified sample proportional to population size. For each of two strata (i.e., all municipalities located in the “Rhein-Neckar District” or in the municipality of the city of “Heidelberg”), individuals or households are drawn in three batches of each three continuous weeks.

For the “Rhein-Neckar District” stratum, a two-stage sample design is used for each batch, within which the primary sampling units (PSU) are drawn as synthetic clusters of secondary sampling units (SSU) (i.e., individuals or households). First, 55 PSUs are randomly selected for each batch independently using probability proportional to size sampling. These 55 PSUs are then distributed across the 56 municipalities (i.e., sampling points) in the “Rhein-Neckar District” stratum using the Cox algorithm. This might result in some sampling points to contain several PSUs. Second, disproportional sampling is used to draw the same number of SSUs for each sampled PSU in order to obtain a self-weighted sample of individuals or households [19]. Since sampling points can contain several PSUs, the number of SSUs per sampling point and batch may vary. As the “Heidelberg” stratum consists only of one municipality, selection of individuals or households is carried out in a single-step simple random sampling process.

The net sample size is then translated into a corresponding gross sample under consideration of the expected response rates in each strategy and the sensitivity and specificity of the pre-screening tool applied to strategies B1 and B2 (see below). The gross sample therefore includes a total of 21,739 individuals or households contacted in the “Rhein-Neckar District” and 6386 in the “Heidelberg” stratum. This way a sample of 28,125 individuals or households is drawn and then randomly allocated to the four strategies A1, A2, B1, and B2 in the ratio 5 to 2.5 to 14 to 7 to eventually yield a final study sample of 2500 saliva tests for each strategy.

### Additional study samples

For the qualitative component, up to 60 in-depth interviews will be conducted with up to 30 study participants (up to 15 in each of the A and B arms) and with up to 30 participation refusers (up to 15 in each of the A and B arms). Both samples are purposefully selected from the quantitative trial sample to ensure all genders and age groups are represented. These samples serve to explore experiences and perceptions of being subjected to health surveillance and study participation. Further, up to 25 asymptomatic SARS-CoV-2-positive study participants are purposefully selected to explore how asymptomatic men and women diagnosed with SARS-CoV-2 give meaning to their diagnosis and to the dialectic between “feeling healthy” while simultaneously having a high likelihood of transmitting the virus to others. In addition, 100 randomly selected trial participants will be sampled to explore study participants’ perspectives on the implementation of each strategy’s screening and testing processes.

### Statistical analyses

The primary analysis will consist of an intent-to-treat (ITT) evaluation. Secondary analysis will include an estimation of the complier average causal effect using standard instrumental variables [20]. Here, the instrumental variable represents the initial random ITT assignment to the four strategies. The effects on continuous primary and secondary endpoints (e.g., cost per fully screened person, cost per confirmed positive case, cost per asymptomatic case, precision of prevalence) are measured using standard ordinary least square regression. For the effects on binary endpoints, we will use the modified Poisson model [21]. Effects on ordinal categorical endpoints (e.g., participant satisfaction measured on a Likert scale) are measured using an ordered logistic regression. All analyses will be adjusted for clustering of standard errors at the level of the randomization unit (i.e., municipalities). Primary analyses will not account for any baseline covariates; secondary analyses will account for the baseline covariates of age and sex.

In order to comprehensively identify the most cost-effective surveillance strategy, the aforementioned ITT analysis is complemented by a cost-effectiveness analysis evaluating the cost-effectiveness of the four surveillance strategies in relation to the status quo of having only passive surveillance. The cost-effectiveness analysis and underlying micro-costing study adopt a health system perspective to account for all costs incurred at the level of the health system, including the economic costs of all resources consumed in relation to the implementation and maintenance of the four active surveillance strategies, as well as the cost of the comparator (i.e., the passive surveillance system). All costs are measured as continuous variables for each individual. To determine the cost-effectiveness of each of the four surveillance strategies, costs and effects (measured as the number of cases detected) will be set into relation to those of the status quo. Incremental cost-effectiveness ratios (ICERs) are computed for each surveillance strategy in relation to the status quo. The strategy with the lowest ICER is considered as most cost-effective and will then be further appraised in conjunction with information on the prognostic models (described below) to orient decision making on the choice of the most suitable surveillance strategy to be implemented in routine practice.

Prognostic models are based on current SARS-CoV-2-related hospitalization numbers and the test results derived from each strategy using a SEIHR (susceptible, exposed, infectious, hospitalized, removed) differential equation. While some parameters for this model are taken directly from the literature, others (e.g., base reproduction number, hospitalization rate) are modeled using a Bayesian approach. Given the rather simplified representation of the SARS-CoV-2 infection process in this model, the predictions will only include data within a defined time window to allow models to react more flexibly to changes in the course of the disease. The inference models are adapted to each strategy. Simulations predict each strategy’s ability to determine the time period in which a new outbreak can be detected with an acceptable probability.

### Power calculation

Based on a dynamic Bayesian prevalence estimate, the successful surveillance strategy is expected to test up to 1000 saliva samples per day in order to generate 2- to 5-week predictions on critical care capacity and other relevant health care parameters in the Rhein-Neckar [22]. Each studied strategy should therefore be able to estimate SARS-CoV-2 prevalence with sufficient accuracy. In May 2020, the 4-week cumulative SARS-CoV-2 prevalence estimated by passive monitoring in the Rhein-Neckar District was around 0.25%. Assuming an equally high prevalence of asymptomatic SARS-CoV-2 carriers, the overall prevalence of asymptomatic and symptomatic carriers was approximately 0.5%. A similar prevalence is assumed for future waves.

Strategy A1 is designed to directly reflect the pandemic events using a purely random sample. With a total of 2500 saliva samples, this strategy is able to detect an overall SARS-CoV-2 prevalence of 0.5% with an accuracy of ±0.35% and a power of 95%; a prevalence of 0.2% would be detected with an accuracy of ±0.25% and a power of 93% (confidence interval according to Agresti-Coull) [23]. Assuming a higher SARS-CoV-2 prevalence within families or households of an infected individual, strategy A2 is expected to detect this higher rate accordingly. Given the upstream pre-screening mechanisms and resulting filter effects in strategies B1 and B2, the resulting detection rates related to these strategies should also differ accordingly.

Sample sizes are therefore designed to yield 2500 saliva samples available for analysis in each study arm. The catchment area of the study (i.e., Rhein-Neckar District plus Heidelberg) has approximately 700,000 inhabitants. To ensure sufficient precision of the measured estimates, a total net case number of 1.4% of the target population (i.e., 0.35% per study arm) is targeted. Based on assumptions on participation rates as well as the sensitivity and specificity of the pre-screening tool, the following gross sample sizes are needed for each strategy:

For strategy A1, a response rate of 50% is assumed after one-time prompting, warranting a gross sample of at least 5000 addresses per batch; for strategy A2, an average household size of two with a response rate of 50% after prompting is assumed, warranting a gross sample of at least 2500 addresses per batch [24]. For strategy B1, a 50% response to the first letter and a 50% response to prompting is assumed; further, a response rate of 80% is assumed for those participating in the pre-screening with positive screening results. To ensure a total of 2500 tested samples, at least 3125 test kits must be mailed out for this strategy. Assuming the pre-screening tool’s sensitivity at 90% and specificity at 70% and given a SARS-CoV-2 prevalence of 0.5%, at least 10,313 (=3125/0.303) participants have to complete the pre-screening. Further assuming a 50% response to the initial pre-screening tool and a 50% response after prompting warrants a gross sample of at least 13,750 addresses. Similar to above, for strategy B2 an average household size of two members is assumed, which warrants a gross sample of at least 6875 addresses. For the entire trial, a minimum gross sample of 28,125 addresses is required.

## Discussion

Once completed, the *CoV-Surv Study* is expected to identify the most effective, acceptable, and optimal surveillance strategy from multiple scientific perspectives, in order to ensure that the most promising strategy is then to be converted into a routine system as needed. This routine system should be scalable to up to 1000 saliva samples per day to allow reliable predictability during the course of a SARS-CoV-2 wave. For its long-term application, meaning once the SARS-CoV-2 pandemic should be successfully controlled and no longer poses an imminent public health risk, the routine system resulting from the *CoV-Surv Study* is further expected to become a stand-by surveillance method that can be quickly reactivated any time in the event of new pandemic threats. As surveillance is based on RT-LAMP testing of biological samples, this test can be quickly adjusted to any other pathogen of public health concern.

### Active vs. passive surveillance

Systems for active surveillance of the general population will gain more importance in the context of pandemics and related disease prevention efforts. As seen during the current SARS-CoV-2 pandemic, the epidemiological course of an easily transmissible illness follows a typical pattern with an initially low overall prevalence in the general population and a comparatively higher prevalence in local clusters. Even in the absence of so-called super-spreader events, infections continue spreading within the general population and therefore lead to increasing overall prevalence rates over time. While complete lockdowns and similarly drastic interventions are effective in reducing the overall prevalence, these measures should only be used over relatively shortly defined time periods. While milder containment methods (e.g., social distancing, mask-wearing, personal hygiene) effectively prevent local outbreaks, they are usually less effective in preventing infection spread.

While useful in identifying positive cases and tracing their potential contacts, passive surveillance strategies should be supplemented by active routine monitoring of larger population groups based on regular testing. Especially in instances where public life is reopened after a more drastic lockdown, active testing frequencies will need to be increased enormously to prevent larger infection chains or super-spreader events to recur, which otherwise would lead to the next lockdown. The pandemic parameters derived from such active surveillance with routine population monitoring therefore not only enable a prospective assessment of the short-term course of a pandemic, but also a more targeted and thus more effective use of local and short-term countermeasures.

### Further considerations

An ideal active surveillance strategy would frequently test every single person in order to identify all asymptomatic and symptomatic carriers as early as possible and to fully prevent further spread. Obviously, such an ideal strategy is unrealistic and active surveillance approaches therefore will have to work with time-varying randomized samples. Estimated prevalence rates will therefore differ in their uncertainty depending on sample sizes used. This will have significant implications on pandemics like the current one. Since COVID-19 is a respiratory disease that is transmitted similarly to most seasonal viral diseases, SARS-CoV-2 infection rates are also likely to increase during winter months. This is expected to lead to an increase in symptoms similar to COVID-19 during these so-called flu seasons, which will further require the density and frequency of SARS-CoV-2 tests will have to be increased to maintain active surveillance.

### Trial status

The final protocol version is “Surveillance_Studienprotokoll_03Nov2020_v1_2” from November 3, 2020. Recruitment started November 18, 2020, and has been completed on December 22, 2020. This protocol was published as a structured summary (https://doi.org/10.1186/s13063-020-04982-z).

## Data Availability

The quantitative data will populate the National COVID-19 Research Network (Nationales COVID-19 Forschungsnetzwerk) and become available in an anonymized form to other researchers. The goal of this research network is to combine all SARS-CoV-2-specific data in Germany to allow for additional joint analyses. This includes plans for the establishment of an overarching data structure based on standardized variable definitions. Researchers outside the network will be asked to follow an application process prior to data access. On a case-to-case basis, requested data is then prepared accordingly, anonymized, and made accessible together with the respective variable dictionary.

## Abbreviations

CoV: Coronavirus
COVID-19: Coronavirus disease caused by SARS-CoV-2
ITT: Intent-to-treat
PSU: Primary sampling unit
RNA: Ribonucleic acid
RT-LAMP: Reverse transcriptase loop-mediated isothermal amplification
RT-PCR: Reverse transcriptase-polymerase chain reaction
RT-qPCR: Reverse transcriptase quantitative polymerase chain reaction
SEIHR: Susceptible, exposed, infectious, hospitalized, removed
SARS: Severe acute respiratory syndrome
SARS-CoV-2: Novel coronavirus causing COVID-19
SSU: Secondary sampling unit
